# Prognostic significance of MRI-based late-course tumor volume in locoregionally advanced nasopharyngeal carcinoma

**DOI:** 10.1186/s13014-022-02087-2

**Published:** 2022-06-27

**Authors:** Ge Yan, Yan Feng, Mingyao Wu, Chao Li, Yiran Wei, Li Hua, Guoqi Zhao, Zhekai Hu, Shengyu Yao, Lingtong Hou, Xuming Chen, Qianqian Liu, Qian Huang

**Affiliations:** 1 Department of Radiation Oncology, Shanghai General Hospital, Shanghai Jiao Tong University School of Medicine, Shanghai, 200080 China; 2 Department of Radiology, Shanghai General Hospital, Shanghai Jiao Tong University School of Medicine, Shanghai, 200080 China; 3 Department of Applied Mathematics and Theoretical Physics, University of Cambridge, Cambridge, CB3 0WA UK; 4 Department of Clinical Neurosciences, University of Cambridge, Cambridge, CB2 0QQ UK; 5 School of Public Health, Shanghai Jiao Tong University School of Medicine, Shanghai, 200025 China; 6 The Comprehensive Cancer Center and Shanghai Key Laboratory of Pancreatic Diseases, School of Medicine, Shanghai General Hospital, Shanghai Jiao Tong University, Shanghai, 201620 China; 7 Cancer Center, Shanghai General Hospital, Shanghai Jiao Tong University School of Medicine, Shanghai, 201620 China

**Keywords:** Locoregionally advanced nasopharyngeal carcinoma (LA-NPC), Late-course volume (LCV), Volume regression rate (VRR), Local recurrence-free survival (LRFS), TNM staging system

## Abstract

**Background:**

To validate tumor volume-based imaging markers for predicting local recurrence-free survival (LRFS) in locoregionally advanced nasopharyngeal carcinoma patients, who underwent induction chemotherapy followed by definitive intensity-modulated radiotherapy.

**Methods:**

We enrolled 145 patients with stage III–IVA nasopharyngeal carcinoma in this retrospective study. Pre-treatment tumor volume (V_pre_) and late-course volume (LCV) were measured based on the MRIs scanned before treatment and during the first 3 days in the sixth week of radiotherapy, respectively. The volume regression rate (VRR) was calculated according to V_pre_ and LCV. Receiver operating characteristic (ROC) curves were used to identify the cut-off best separating patient subgroups in assessing the prognostic value of V_pre,_ LCV and VRR. The Kaplan–Meier method was used for survival analysis. Prognostic analyses were performed using univariate and multivariate COX proportional hazard models.

**Results:**

The LCV was 5.3 ± 0.5 (range 0–42.1) cm^3^; The VRR was 60.4 ± 2.2% (range 2.9–100.0). The median follow-up period was 36 months (range 6–98 months). The cut-off value of LCV determined by the ROC was 6.8 cm^3^ for LRFS prediction (sensitivity 68.8%; specificity 79.8%). The combination of LCV and VRR for LRFS prediction (AUC = 0.79, *P* < 0.001, 95% CI 0.67–0.90), LCV (AUC = 0.74, *P* = 0.002, 95% CI 0.60–0.88) and V_pre_ (AUC = 0.71, *P* = 0.007, 95% CI 0.56–0.85) are better than T category (AUC = 0.64, *P* = 0.062, 95% CI 0.50–0.79) alone. Patients with LCV ≤ 6.8 cm^3^ had significantly longer LRFS (*P* < 0.001), disease-free survival (DFS, *P* < 0.001) and overall survival (OS, *P* = 0.005) than those with LCV > 6.8 cm^3^*.* Multivariate Cox regression showed LCV was the only independent prognostic factor for local control (HR = 7.80, 95% CI 2.69–22.6, *P* < 0.001).

**Conclusions:**

LCV is a promising prognostic factor for local control and chemoradiosensitivity in patients with locoregionally advanced NPC. The LCV, and the combination of LCV with VRR are more robust predictors for patient survival than T category.

## Background

Nasopharyngeal carcinoma (NPC) is a unique head and neck cancer, highly prevalent in Southern China and other Southeast Asian countries but less common in other regions [1]. Radiotherapy (RT) is the mainstay of NPC treatment due to the challenge in surgical resection and radiosensitivity. For locoregionally advanced NPC (LA-NPC) patients, induction chemotherapy (IC) followed radiotherapy is widely accepted as the standard because of the dismal prognosis [2].

The TNM staging system is widely used for clinical decision and prognosis for NPC [3]. However, patients of the same stage frequently display distinct clinical characteristics and outcomes, albeit receiving the identical treatment [4]. Evidence suggests that the T staging category, mainly based on anatomical locations, is limited in predicting local control, as it fails to consider local tumor burden [5, 6]. In the era of intensity-modulated radiotherapy (IMRT), the local control rate for NPC has dramatically improved [7], which, however, may further decrease the value of the staging parameters for predicting local failure [8]. Particularly, the prognostic value of the T category is weaker than the previous. Even though modifications have been made in the updated staging system, it is still challenging to predict local control purely based on this system [9].

Different with other head and neck carcinomas, the T category in NPC has not included the tumor diameter as a staging variable. This is primarily due to the difficulty in accurately measuring the tumor, particularly for the tumors with fewer regular shapes and more frequent skull base involvement. Tumor volume is long recognized as an indicator of tumor burden and RT response in many types of cancer [10]. With the development of imaging techniques, it becomes more feasible to quantify tumor volume precisely based on MRI.

The majority studies focused on the pre-treatment tumor volume. A previous study found that patients with larger primary tumor volume were associated with significantly poorer local control and disease-specific survival. Moreover, only the primary tumor volume was found an independent factor in predicting local control [11]. Another study reported that primary gross tumor volume was significantly associated with locoregional control, distant metastasis, and overall survival for patients with LA-NPC [12].

Recently, increasing importance has been attached to the post-treatment tumor volume for prognostication. Late-course volume (LCV) represents the tumor burden of LA-NPC after induction chemotherapy, near the end of definitive IMRT, which theoretically reflects the treatment sensitivity. Radiation oncologists generally assess radiotherapy response and adjust the final radiation dose according to the MRI at this point. Therefore, it is crucial to evaluate LCV and volume regression rate (VRR) based on MRIs.

In this study, we sought to develop more robust and accurate volume-based predictors for more precise treatment planning in LA-NPC patients. We hypothesized that LCV and VRR of tumors during radiotherapy based on MRIs could provide more crucial value for evaluating treatment response and prognosis of LA-NPC.

## Methods

### Patients

**Fig.1:**
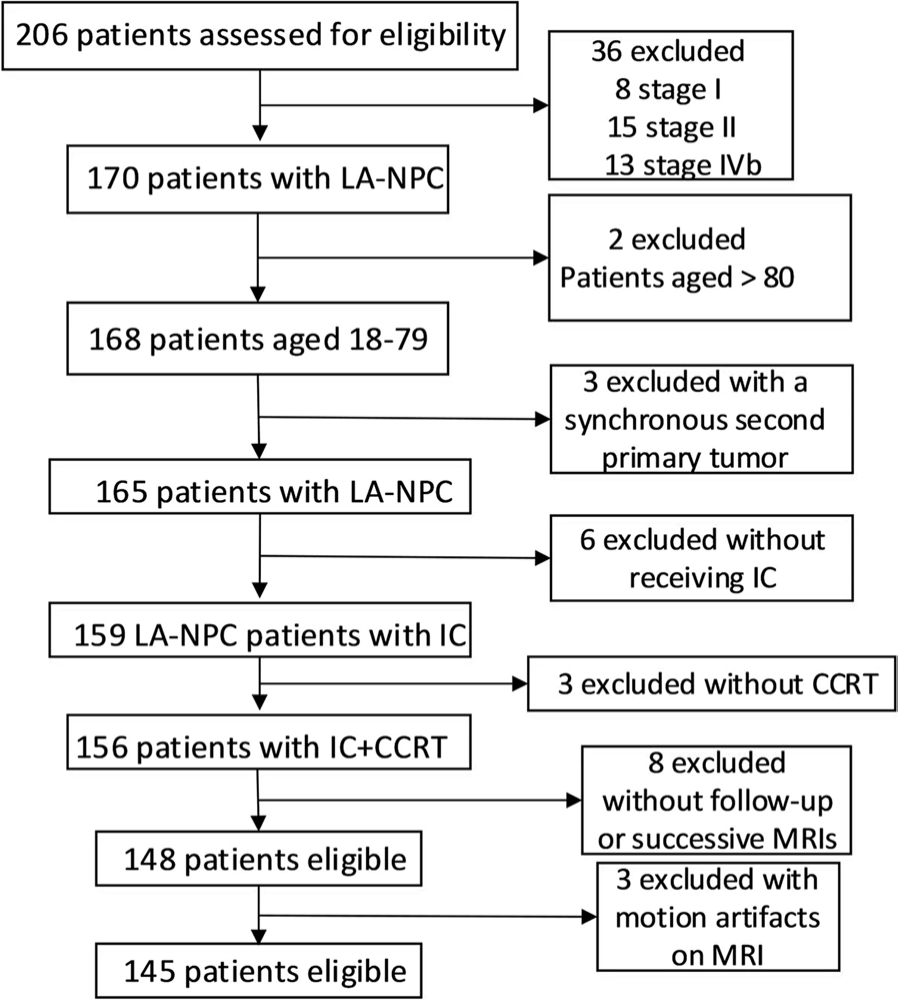
Patient inclusion diagram. *LA-NPC* locoregionally advanced-nasopharyngeal carcinoma, *IC* induction chemotherapy, *CCRT* concurrent chemoradiotherapy

This study initially included 206 newly diagnosed consecutive NPC patients from January 2012 to August 2019 in our institution. All patients underwent biopsy and pathological examination for NPC diagnosis and treatment. All imaging data was reviewed, and T and N categories were reclassified according to the eighth edition of AJCC (American Joint Committee on Cancer) TNM staging system. Among the 206 patients, a total of 61 patients were excluded according to the predefined exclusion criteria: (1) 8 stage I patients, 15 stage II patients, and 13 stage IVB patients; (2) 2 patients aged > 80; (3) 3 patients with a synchronous second primary tumor; (4) 6 patients without receiving induction chemotherapy; (5) 3 patients without concurrent chemoradiotherapy (CCRT); (6) 8 patients without follow-up or successive MRIs; (7) 3 patients with motion artifacts on MRI (Fig. 1). This retrospective study was approved by the Institutional Review Board of Shanghai General Hospital, Shanghai Jiao Tong University School of Medicine, China (No.2019062). Informed consent was obtained from all the patients.

### Diagnosis and treatment

Pre-treatment evaluations included a complete history and physical examination emphasizing the head and neck area, a complete blood count, a serum biochemical profile, and liver/renal function tests. Nasopharyngeal contrast-enhanced MRI, neck contrast-enhanced MRI or contrast-enhanced CT, chest and abdomen contrast-enhanced CT or sonography, emission computed tomography (ECT) or optional whole-body 18 F-fluorodeoxyglucose (FDG) PET/CT was performed. All patients were treated by the multidisciplinary team, including head and neck surgeons, radiation oncologists, medical oncologists, radiologists, pathologists and dieticians.

### Chemotherapy Regimen

All patients received induction chemotherapy. IC was administered for two cycles every 3 weeks. Of the 145 patients, 140 patients completed two cycles of induction chemotherapy. Among the patients withdrawn after the first cycle, two patients were due to the side effect on liver function (Grade 2), and three patients quit without consent to further induction chemotherapy. The regimen of IC consisted of docetaxel (75 mg/m^2^ on day 1), cisplatin (75 mg/m^2^ on day 1), and 5-fluorouracil (2500 mg/m^2^ as an intravenous infusion over 120 h) or Capecitabine (1000 mg/m^2^/d, days 1–14). The concurrent chemotherapy regimen consisted of weekly cisplatin (40 mg/m^2^) or 3-weekly cisplatin (100 mg/m^2^) during radiotherapy, which began on the first day of radiotherapy as planned.

### Radiotherapy

All patients were immobilized with a tailored head-shoulder thermoplastic mask in the supine position and treated using IMRT, delivered within 3 weeks after induction chemotherapy. The high-risk clinical target volume (CTV1) consisted of the gross tumor volume (GTV) with an expansion of 5 mm, the entire nasopharyngeal cavity, anterior one-third of the clivus (the whole clivus if invaded), the skull base, the pterygoid plates, the parapharyngeal space, the inferior sphenoid sinus (the whole sphenoid sinus had to be covered for T3 and T4 disease), posterior one-third of the nasal cavity and the maxillary sinus, and drainage of the upper nodal region, including retropharyngeal lymph nodes and levels II, III, and V(a). The low-risk CTV (CTV2) included level IV and V(b) nodal regions in patients without metastatic cervical lymph nodes. For patients of the N3 stage, all the neck levels from II to V were defined as CTV1. The planning target volumes (PTV) of the primary nasopharynx tumor volume, including retropharyngeal lymph nodes (PTV-P) and positive lymph nodes (PTV-LN) received 66–70.4 Gy in 30–32 fractions, whereas 60 and 54 Gy in 30–27 fractions were prescribed to the PTVs of CTV1 and CTV2. Radiation therapy was delivered over one fraction daily, 5 days per week. The treatment planning optimization and evaluation were based on the radiation therapy oncology group (RTOG) 0225 trial [13].

### MRI protocols

MRI scanning was performed on a 3.0 T scanner (Discovery MR 750w, GE Medical Systems, USA/Ingenia, Philips Medical Systems, The Netherlands) with a head-and-neck combined coil. The following sequences were performed: an axial T1WI-weighted sequence (TR/TE = 473/11 ms, slice thickness = 4 mm, spacing between slices = 5 mm, field of view = 200 × 200 mm^2^, flip angle = 90°), an axial fat-suppressed T2-weighted sequence (TR/TE = 3300/70 ms, slice thickness = 4 mm, spacing between slices = 5 mm, field of view = 200 × 200 mm^2^, flip angle = 90°), and a fat-suppressed T1-weighted sequence in the axial and coronal planes following a bolus injection of 0.2 ml/kg gadopentetate dimeglumine (Magnevist, Schering, Berlin, Germany), then contrast-enhanced T1-weighted (CET1-w) images were obtained.

Scanning for LCV was performed on any day during the first 3 days in the sixth week of radiation therapy, i.e., the day between 26 and 28th exposure to radiation.

### Tumor volume measurement

Both primary tumor and retropharyngeal lymph nodes were included in measuring pre-treatment tumor volume (V_pre_) and LCV. VRR was determined using the equation VRR (%) = (V_pre_ − LCV) × 100/V_pre._ Tumor regions of interest (ROIs) were manually delineated on the co-registered axial CET1-w and T2-w images by two radiation oncologists (G.Y., 12 years of experience and M.Y.W., 5 years of experience) and a radiologist (Y.F., 12 years of experience), independently. All the raters received initial training following the same standard and were blinded to the clinical outcomes. The concordance of the two raters was assessed by the DICE score. For the case of disagreement, the consensus was achieved after discussion among the three raters. All the raters received initial training and were blinded to the clinical outcomes. The delineation was done using the 3D slicer v4.6.219 (Surgical Planning Laboratory, Brigham and Women’s Hospital, Boston, Massachusetts; www.slicer.org). The raw volumes of ROIs were calculated using the function of fslmaths in FSL[14].

### Follow up and endpoint

All patients were followed up every 1–3 months during the first 2 years, every 6 months in year 2–5, and annually thereafter. We defined the survival time as the first day of IC until the target event or last follow-up visit (censored). The primary endpoint was defined as local recurrence-free survival (LRFS, persistence/recurrence at nasopharynx). The secondary endpoints were defined as disease-free survival (DFS, staying free of disease after treatment) and overall survival (OS, death due to any cause).

### Statistical analysis

All statistical analyses were performed using the SPSS (version 20.0; Chicago, IL, USA). The LCV, VRR and the clinicopathological parameters (gender, age, T category, N category, TNM staging and WHO histological type) were compared using t-test, Mann–Whitney U test, or Kruskal–Wallis test, as appropriate. Kaplan–Meier and Cox proportional hazard regression analyses were performed to assess patients’ survival. For the Kaplan–Meier analysis, the differences were compared using the log-rank test. For the multivariate Cox regression analysis, we used forward selection to test the independent significance of different factors. Receiver operating characteristic (ROC) curves were performed to identify the cut-off values for the primary endpoint. The areas under the ROC curve (AUC) were used to evaluate the prognostic value of LCV and VRR. Two-sided *P* < 0.05 were considered to indicate statistical significance.

## Results

### General clinical characteristics and follow-up

We included 145 eligible patients (median age 54 years old, range 27–76 years old, 33 or 22.8% females) in the final analysis (Table 1). Among them, 128 (88.3%) patients were diagnosed as non-keratinizing undifferentiated NPC, i.e., the World Health Organization (WHO) pathological type III. All patients were restaged using the eighth edition TNM staging system, and 112 (77.2%) patients were classified as stage III, and 33 (22.8%) as stage IVA, respectively. All patients underwent CCRT after IC. The mean pre-treatment tumor volume was 13.6 ± 1.0 cm^3^ (range 0.8–67.7 cm^3^; median, 10.4 cm^3^), and the mean LCV was 5.3 ± 0.5 cm^3^ (range 0–42.1 cm^3^; median, 3.3 cm^3^). Across all patients, the VRR was 60.4 ± 2.2% (range 2.9–100.0; median, 64.6%).

**Table 1:** Clinical Characteristics of the 145 patients with LA-NPC

Characteristics	No. of patients	Percentage (%)	LCV ≤ 6.8 cm^3^ (n = 108)	LCV > 6.8 cm^3^ (n = 37)	*P* value
Age (years)			52.9 ± 12.2	53.4 ± 11.4	0.591
< 55	72	49.7			
≥ 55	73	50.3			
Gender					0.519
Male	112	77.2	82	30	
Female	33	22.8	26	7	
T category					< 0.001
T1	13	9.0	13	0	
T2	66	45.5	57	9	
T3	48	33.1	29	19	
T4	18	12.4	9	9	
N category					0.397
N0	6	4.1	5	1	
N1	17	11.7	10	7	
N2	105	72.4	81	24	
N3	17	11.7	12	5	
TNM staging					0.011
III	112	77.2	89	23	
IVA	33	22.8	19	14	
Histological type					0.219
I	3	2.1	3	0	
II	14	9.7	12	2	
III	128	88.3	93	35	
Vpre					
≤ 16.3 cm^3^	108	74.5	57.4 ± 6202.0	118.5 ± 4383.0	< 0.001
> 16.3 cm^3^	37	25.5			

*V*_*pre*_ tumor volume before treatment, *LCV* late-course volume; *P* value indicates the difference in the c-indexes

Unitl April 2020, the median follow-up was 36 months (range 6–98 months). Of the 145 patients, 16 (11%) developed local recurrence, 21 (14.5%) developed distant metastasis. There were 30 (20.7%) deaths: 11 (7.6%) died of relapse, 10 (6.9%) died of metastasis, 4 (2.7%) died of both locoregional relapse and metastasis, and 5 (3.4%) died of other diseases. The cut-off value of LCV determined by the ROC was 6.8 cm^3^ for LRFS prediction (sensitivity 68.8%; specificity 79.8%); V_pre_ was 16.3 cm^3^ for LRFS prediction (sensitivity 62.5%; specificity 79.1%) and VRR was 75.1% for LRFS prediction (sensitivity 93.8%; specificity 39.5%).

### Comparison of ROC curves among T category, LCV and/or VRR for LFRS prediction

**Fig. 2:**
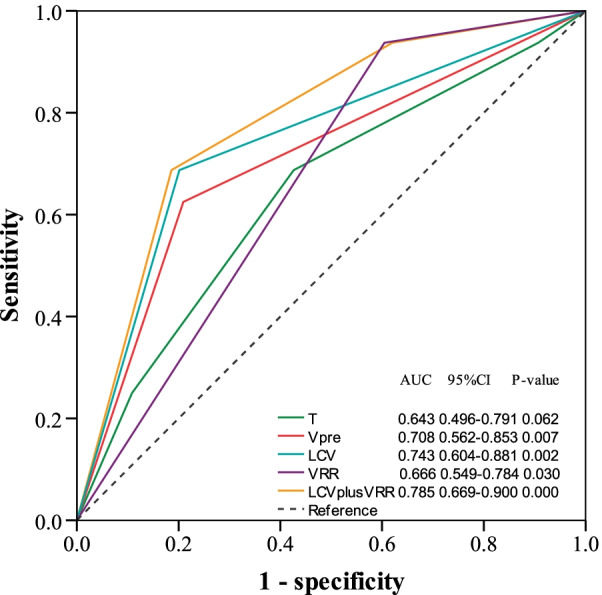
ROC curve to show the AUC of T category, V_pre_, LCV, VRR and LCV plus VRR for LRFS. *ROC* receiver operating characteristic, *AUC* area under the curve, *V*_*pre*_ tumor volume before treatment, *LCV* late-course volume, *VRR* volume regression rate, *LRFS* local recurrence-free survival, *CI* confidence interval; *P* value indicates the difference in the c-indexes

**Fig. 3:**
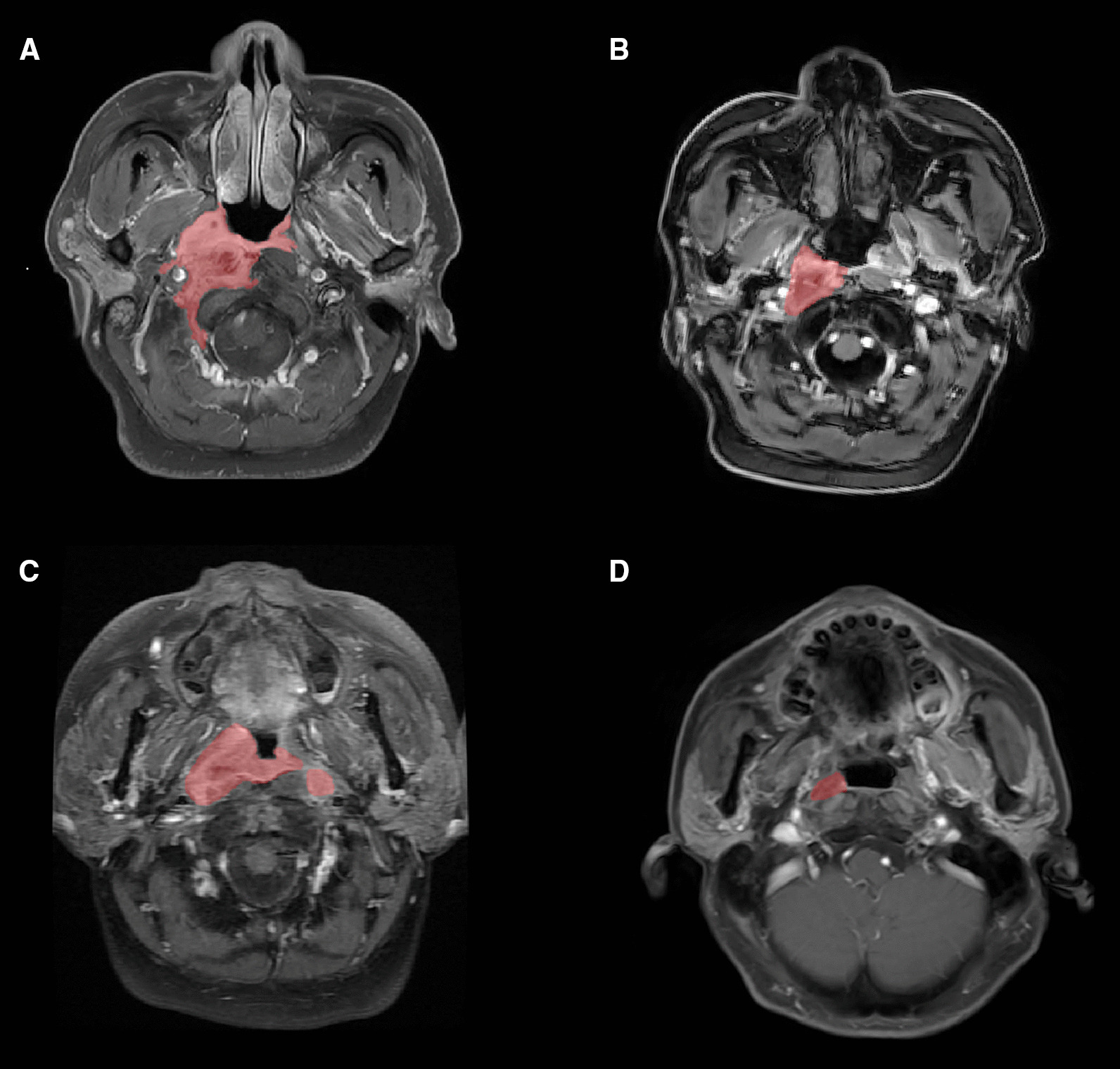
Contrast axial T1-weighted MRI obtained in pre-treatment (**A**, **C**) and late-course of IMRT after IC (**B**, **D**). ROIs were delineated to indicate the Vpre (**A**, **C**, red) and LCV (**B**, **D**, red). Case 1: a 43-years of male with T3N2M0 NPC, showing a VRR < 75.1%, from Vpre = 30.2 cm^3^ (**A**) to LCV = 22.4 cm^3^ (**B**), suffered from local recurrence at the 28th month of follow-up. Case 2: a 40-years of male with T4N2M0 NPC, showing a VRR > 75.1%, from Vpre = 21.2 cm^3^ (**C**) to LCV = 1.7 cm^3^ (**D**), without local recurrence till the final follow-up (at the 81th month). *IMRT* intensity-modulated radiotherapy, *IC* induction chemotherapy, *ROI* tumor regions of interest, *NPC* nasopharyngeal carcinoma, *Vpre* tumor volume before treatment, *LCV* late-course volume, *VRR* volume regression rate

Patients were divided into two groups according to the cut-off value of LCV for LRFS (≤ 6.8 cm^3^ or > 6.8 cm^3^). The comparison of ROC curves showed the combination of LCV and VRR, or LCV, Vpre and VRR alone, is better than the T category in LRFS prediction (Fig. 2). The lower LCV and higher VRR are associated with better local response (Fig. 3).

### Prognostic factors

**Fig. 4:**
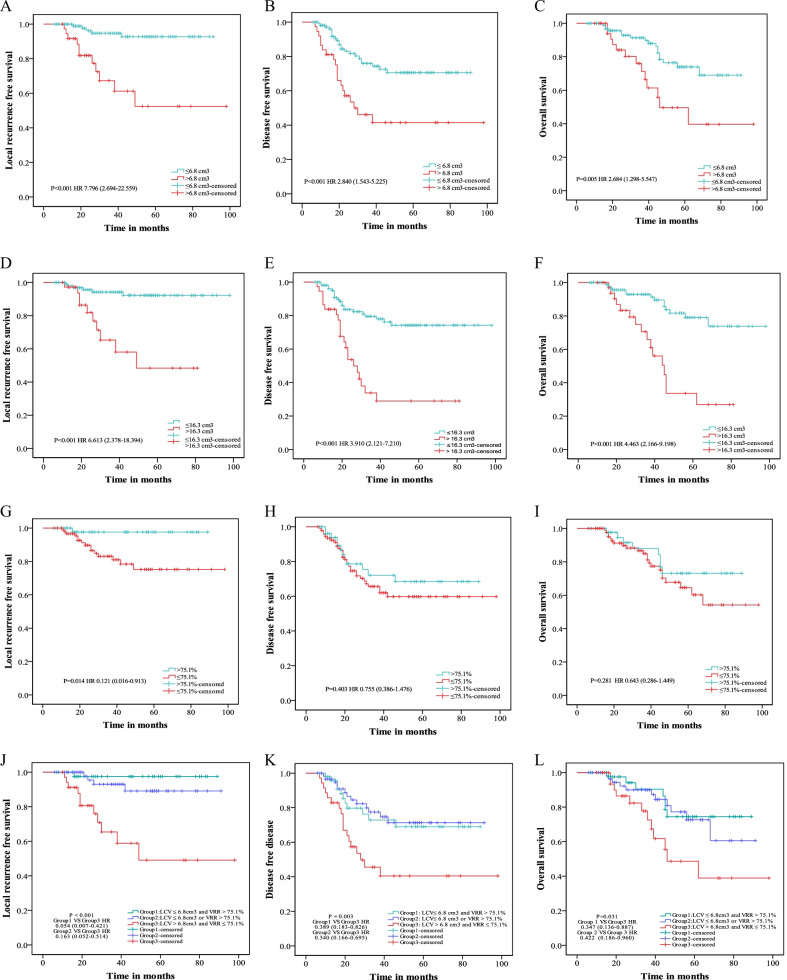
Kaplan–Meier survival curves for patients with LA-NPC of LRFS (**A**,** D**,** G**,** J**), DFS (**B**,** E**,** H**,** K**), and OS (**C**,** F**,** I**,** L**) for LCV, V_pre_, VRR and LCV plus VRR. *LA-NPC* locoregionally advanced nasopharyngeal carcinoma, *V*_*pre*_ tumor volume before treatment, *LCV* late-course volume, *VRR* volume regression rate, *LRFS* local recurrence-free survival, *DFS* disease-free survival, *OS* overall survival, *HR* hazard ratio; *P* Value indicates the difference in the c-indexes

The patients with lower LCV (≤ 6.8 cm^3^) demonstrated significantly better LRFS (*P* < 0.001), DFS (P < 0.001), OS (*P* = 0.005) than those with higher LCV (> 6.8 cm^3^) (Fig. 4A–C). We also used the identified cut-offs of V_pre_ and VRR (16.3 cm^3^ for V_pre_ and 75.1% for VRR) to evaluate the risks of LRFS, DFS and OS. We observed that higher V_pre_ was significantly associated with worse LRFS (*P* < 0.001), DFS (*P* < 0.001) and OS (*P* < 0.001) (Figs. 3 and 4D, E). Of note, VRR was the only significant factor for LRFS (*P* = 0.005) (Fig. 4G–I). Subsequently, we divided patients into three groups according to the cut-offs of LCV and VRR. Our results showed that LCV plus VRR was significant for LRFS (*P* < 0.001), DFS (*P* = 0.003) and OS (*P* = 0.031) (Fig. 4J–L). The multivariate analysis (Table 2) showed LCV was the only independent prognostic factor for local control.

**Table 2:** Univariate and multivariate cox proportional hazards regression analysis of potential risk factors for endpoints

Variables	Category	Univariate									Multivariate								
		LRFS			DFS			OS			LRFS			DFS			OS		
		HR	95%CI	*P*	HR	95%CI	*P*	HR	95%CI	*P*	HR	95%CI	*P*	HR	95%CI	*P*	HR	95%CI	*P*
Age	< 55 versus ≥ 55	1.383	0.515–3.718	0.518	1.008	0.550–1.848	0.978	0.981	0.479–2.010	0.958	–	–	–	–	–	–	–	–	–
Gender	Male versus female	2.248	0.511–9.983	0.270	2.365	0.929–6.019	0.061	1.523	0.583–3.982	0.384	–	–	–	–	–	–	–	–	–
T	1,2 versus 3,4	0.350	0.121–1.010	0.042	0.528	0.285–0.979	0.038	0.504	0.239–1.062	0.065	–	–	–	–	–	–	–	–	–
N	0,1 versus 2,3	0.727	0.165–3.210	0.672	0.868	0.365–2.063	0.747	1.057	0.429–2.606	0.903	–	–	–	–	–	–	–	–	–
TNM	III versus IVA	0.588	0.204–1.696	0.320	0.393	0.210–0.735	0.002	0.387	0.185–0.811	0.009	–	–	–	–	–	–	–	–	–
Vpre	> 6.8 cm^3^ versus ≤ 6.8 cm^3^	6.613	2.378–18.394	< 0.001	3.910	2.121–7.210	< 0.001	4.463	2.166–9.198	< 0.001	–	–	–	3.910	2.121–7.210	< 0.001	4.463	2.166–9.198	< 0.001
LCV	> 16.3 cm^3^ versus ≤ 16.3 cm^3^	7.796	2.694–22.559	< 0.001	2.840	1.543–5.225	< 0.001	2.684	1.298–5.547	0.005	7.796	2.694–22.559	< 0.001	–	–	–	–	–	–
VRR	> 75.1% versus ≤ 75.1%	0.121	0.016–0.913	0.014	0.755	0.386–1.476	0.403	0.643	0.286–1.449	0.281	–	–	–	–	–	–	–	–	–

*V*_*pre*_ tumor volume before treatment, *LCV* late-course volume, *VRR* volume regression rate, *LRFS* local recurrence-free survival, *DFS* disease-free survival, *OS* overall survival, *HR* hazard ratio from Cox hazards model, *CI* confidence interval; *P* Value indicates the difference in the c-indexes. The following parameters were included in the models as covariates for each analysis: age, gender, T category, N category, TNM staging, V_pre_, LCV and VRR

## Discussion

It is recognized that the TNM staging system fails to predict prognosis consistently for locoregionally advanced NPC [15, 16]. These limitations could be mainly due to the heterogeneity of NPC, where at least 20% of the patients showed poor effect, even though patients with the same TNM stage underwent similar treatment regimens [17, 18]. Moreover, with the prevalence of IMRT, the prognostic value of staging parameters, especially the T category, has been challenged [8]. In this study, we identified several significant factors in a retrospective, well-characterized NPC dataset, with two time-point MRIs and standardized treatment regimens. Both LCV and VRR showed significant effects on local control. Particularly, LCV was an independently prognostic factor for LRFS.

Additionally, LCV was a significant factor for DFS and OS. We identified the cut-offs of 6.8 cm^3^ for LCV and 75.1% for VRR in predicting LRFS. LCV is better in predicting LRFS than V_pre_, T category, or VRR. The combination of LCV with VRR using their thresholds was more robust than any of LCV, VRR, V_pre_ or T category alone in predicating LRFS.

Although tumor volume is not a factor included in the TNM staging system of NPC, the prognostic significance of tumor volume before treatment has been studied and well demonstrated in multiple studies [12, 19, 20]. Our study obtained similar results about V_pre_ in LA-NPC. Further, we found its specificity and sensitivity is better than T category for LRFS.

Recently, the importance of post-treatment tumor volume for prognostication is increasingly recognized. Some studies focused on tumor volume of post induction chemotherapy. It is showed that post-IC tumor volume was a robust variable to reflect tumor burden and had utility for prognostication [21]. Other studies focused on tumor volume after radiotherapy, which demonstrated that the residual tumor volume (RTV) after the first treatment (detected at the 6th month control after completion of first treatment) was a significant prognostic factor for local regional recurrence-free survival (LRRFS) [22]. Nonetheless, the proposition of using tumor volume in the late radiation course and VRR for prognostication in LA-NPC is neglected. LCV represents the tumor burden of LA-NPC after IC and near the end of definitive radiotherapy, and theoretically reflects the sensitivity of NPC to radiation.

Tumor burden on local control using radiotherapy has been evaluated as a predictor because of the association between large volume and adverse biological factors, including clonogen number, hypoxia, and radioresistance [20]. Nevertheless, the T category is only based on anatomical locations and fails to consider local tumor burden and heterogeneity. NPC patients normally need another MRI on any day between the 26th and the 28th exposure to radiation to evaluate radiation response and tumor burden. According to this MRI, a final total dose of radiotherapy will be adjusted and the following treatment plans will be made. We defined and measured LCV using this time-point MRI. LCV is near the end of IC and CCRT, which should be better to predict local control and radiation sensitivity after the standard treatment for LA-NPC.

To the best of our knowledge, this study is the first attempt to evaluate both tumor volume and volume regression rate at late-course radiotherapy for LRFS in patients with LA-NPC. Importantly, we enrolled the patients with the consistent protocol of IC plus CCRT and two time-points contrast-enhanced MRIs. The results for predicting LRFS in this study have clinical significance. The potential clinical utility of LCV and VRR for prognostication and decision-making guidance is more critical and practical than the T category and V_pre_. But some limitations must be taken into account. Firstly, this is a retrospective study, and the results need further validation of prospective studies. Secondly, our data is from a single center, and therefore, external validation with datasets from other centers is needed. Thirdly, EBV-DNA copy number is not included as a covariate in the multivariate analysis due to limited available data. Lastly, the sample size of the current study is relatively small.

## Conclusions

In conclusion, in this retrospective study, we found that LCV and VRR were significantly prognostic factors for local control and radiation sensitivity in patients with LA-NPC. Further investigations are needed to explore heterogeneity and radiosensitivity of LA-NPC, using ROIs based on these two time-point MRIs.

## Data Availability

The datasets used and analyzed during the current study are available from the corresponding author on reasonable request.

## Abbreviations

AJCC: American Joint Committee on Cancer
AUC: Areas under the receiver operating characteristic curve
CCRT: Concurrent chemoradiotherapy
CET1-w: Contrast-enhanced T1-weighted
CTV1: High-risk clinical target volume
CTV2: Low-risk clinical target volume
DFS: Disease-free survival
ECT: Emission computed tomography
FDG: Fluorodeoxyglucose
GTV: Gross tumor volume
IC: Induction chemotherapy
IMRT: Intensity-modulated radiotherapy
LA-NPC: Locoregionally advanced nasopharyngeal carcinoma
LCV: Late-course volume
LRFS: Local recurrence-free survival
LRRFS: Local regional recurrence-free survival
NPC: Nasopharyngeal carcinoma
OS: Overall survival
RT: Radiotherapy
PTV: Planning target volume
ROC: Receiver operating characteristic curves
ROIs: Regions of interest
RTOG: Radiation Therapy Oncology Group
RTV: Residual tumor volume
Vpre: Pre-treatment tumor volume
VRR: Volume regression rate
